# Materials-Related Aspects of Thermochemical Water and Carbon Dioxide Splitting: A Review

**DOI:** 10.3390/ma5112015

**Published:** 2012-10-24

**Authors:** Martin Roeb, Martina Neises, Nathalie Monnerie, Friedemann Call, Heike Simon, Christian Sattler, Martin Schmücker, Robert Pitz-Paal

**Affiliations:** 1 Institute of Solar Research, German Aerospace Center (DLR), Köln 51170, Germany; E-Mails: martina.neises@dlr.de (M.N.); nathalie.monnerie@dlr.de (N.M.); friedemann.call@dlr.de (F.C.); christian.sattler@dlr.de (C.S.); robert.pitz-paal@dlr.de (R.P.-P.); 2 Institute of Material Research, German Aerospace Center (DLR), Köln 51170, Germany; E-Mails: heike.simon@dlr.de (H.S.); martin.schmuecker@dlr.de (M.S.)

**Keywords:** water splitting, CO_2_- splitting, thermochemical cycle, redox material, sulfur cycle, solar power, CO, solar fuels, hydrogen

## Abstract

Thermochemical multistep water- and CO_2_-splitting processes are promising options to face future energy problems. Particularly, the possible incorporation of solar power makes these processes sustainable and environmentally attractive since only water, CO_2_ and solar power are used; the concentrated solar energy is converted into storable and transportable fuels. One of the major barriers to technological success is the identification of suitable active materials like catalysts and redox materials exhibiting satisfactory durability, reactivity and efficiencies. Moreover, materials play an important role in the construction of key components and for the implementation in commercial solar plants. The most promising thermochemical water- and CO_2_-splitting processes are being described and discussed with respect to further development and future potential. The main materials-related challenges of those processes are being analyzed. Technical approaches and development progress in terms of solving them are addressed and assessed in this review.

## 1. Introduction

Hydrogen produced from renewable resources is considered a key element of future energy technology and economy. For that purpose, the product needs to be carbon-lean or, even better, carbon-free, meaning that the raw material is water and the energy source is renewable. Solar electricity—generated via photovoltaics (PV) or concentrating solar power (CSP)—followed by electrolysis of water at low temperature, is a viable technical route for producing H_2_. Today, it can be considered as a benchmark for other routes such as solar-driven thermochemical water splitting cycles that offer the potential of energy efficient large-scale production of hydrogen. The electricity demand for electrolysis can be significantly reduced if the electrolysis of water proceeds at higher temperatures (1073–1273 K via Solid Oxide Electrolyzer Cells (SOEC)). Concentrated solar energy can be applied to provide the high-temperature process heat.

The single-step thermal dissociation of water is, at a first glance, the simplest reaction to split water. However, because of its unfavorable thermodynamics, the process is one of the most challenging with respect to practical realization. Although water thermolysis is conceptually simple, the need for a high-temperature heat source above 2500 K to achieve a reasonable degree of dissociation and the requirement for an effective technique to separate H_2_ and O_2_ at high temperatures to avoid an explosive mixture of these two gases are major barriers to technical success.

Water splitting thermochemical cycles avoid the separation problem and further allow operation at moderately high temperatures. The screening and search of appropriate thermochemical cycles started in the 1960s and the number of theoretical candidates was immense. Therefore, during the 1970s and early 1980s, many studies and comparisons were carried out to identify the most promising cycles based on different criteria such as thermodynamics, theoretical efficiencies, and projected cost [1,2,3,4]. In general, most of the development effort applied during those years was promoted by research institutions and industry from the nuclear energy sector, with the intention to diversify the use of thermal energy supplied by nuclear reactors. To this end, the programs developed by the Joint Research Centre of the European Union in Ispra, Italy [2], by General Atomics [5] and Westinghouse [6] in the United States, and by the Japanese Atomic Energy Research Institute [7] are particularly worthy of mention.

In the late 1980s, the interest in thermochemical cycles decreased drastically. Since then until the late 1990s, only marginal progress was reported mainly on the UT-3 cycle [8] developed by and named after the University of Tokyo and on the sulfur–iodine cycle originally proposed and named after the company General Atomics [9]. A revival in the research and development (R and D) of thermochemical cycles has taken place in the past few years. The driving force is the production of hydrogen as a greenhouse gas-free energy carrier to fulfill the requirements of the Kyoto Protocol.

Previous studies performed on thermochemical cycles were mostly characterized by using process heat at temperatures below 1223 K, which are expected to be available in the future from very high temperature nuclear reactors (VHTR). These cycles require three or more chemical reaction steps (two steps in the case of the hybrid sulfuric acid cycle incorporating one electrolytic step) and are challenging because of material problems and inherent inefficiencies associated with heat transfer and product separation at each step. The leading candidates for multistep thermochemical cycles include mainly a three-step sulfur iodine cycle based on the thermal decomposition of sulfuric acid at 1123 K and a four-step UT-3 cycle based on the hydrolysis of calcium and iron bromide at 1023 K and 873 K, respectively.

Recently, the so-called CuCl cycle [10] has attracted a lot of attention, since the maximum process temperature is less than 823 K, which allows powering it by heat from less advanced nuclear reactors [11]. It is also interesting for solar hydrogen productions since the mentioned temperature level even can be reached by advanced parabolic trough or linear Fresnel technology [12].

Specific attention should be drawn to solar thermochemical water splitting by means of suitable redox reactions since most of them only apply two process steps; these are the so-called redox cycles. In this concept, an ignoble metal or multivalent metal oxide is employed which allows water decomposition at moderate temperatures, whereby the metal is oxidized or the multivalent metal oxide approaches a higher oxidation state, respectively. The water splitting agent can be regenerated later on at high temperatures driven by solar thermal energy and by low oxygen partial pressure. Then a lower oxidation state arises and, hence, the system is ready for the next water-splitting step. Water splitting by redox reactions essentially is a closed loop process with water and heat energy as inputs, and hydrogen and oxygen as outputs. A comprehensive overview on such redox based thermochemical cycles is given by Abanades *et al.* [13].

**Figure 1:**
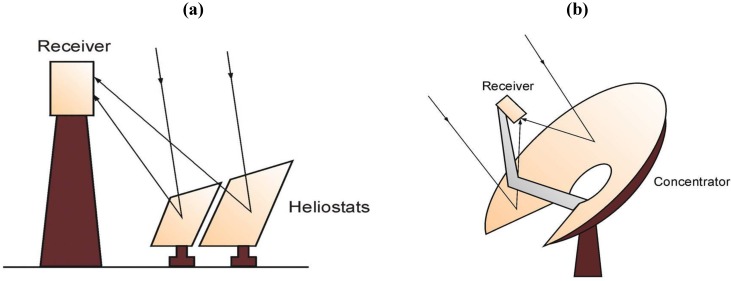
Schematic of (**a**) a central receiver system and (**b**) a solar dish system.

For all of the aforementioned processes, the heat to drive the chemical reaction can be provided through the concentration of direct solar irradiation with optical systems, e.g., mirrors. Due to the high temperatures required for solar thermal water splitting, only concentrating solar technologies working with a point focusing system can provide the necessary process temperatures with high efficiency. Such systems are parabolic dish systems or central receiver systems shown in Figure 1. The chemical reactor can be placed in the focus of the system and the radiation either enters the reactor through a quartz window or is then directly absorbed by the chemical reactants or it is absorbed on a black surface, for example by tubes, and transferred to the reactants by convection and conduction [14].

Materials are a key issue of solar thermal water splitting [13,15,16]. The role of material science is not limited to providing suitable redox agents, but is also focused on the microstructural stability of the employed substances, on reaction kinetics and on kinetics of atomic diffusion and the type and rate of transformation on catalysts’ activity and stability. Moreover, high temperature water-splitting processes require suitable materials for substrates and containments being stable against the reaction system and environmental influences, in particular if considering the harsh thermal conditions and chemical atmospheres they have to face. In this context, also solar absorbance and resistance against thermal shock and fatigue must be considered. Those aspects will be highlighted and analyzed for the most prominent thermochemical cycles in the following chapters. As well reactor technologies developed for the different thermochemical processes will be shown.

## 2. Metal Oxide-Based Redox Materials

**Figure 2:**
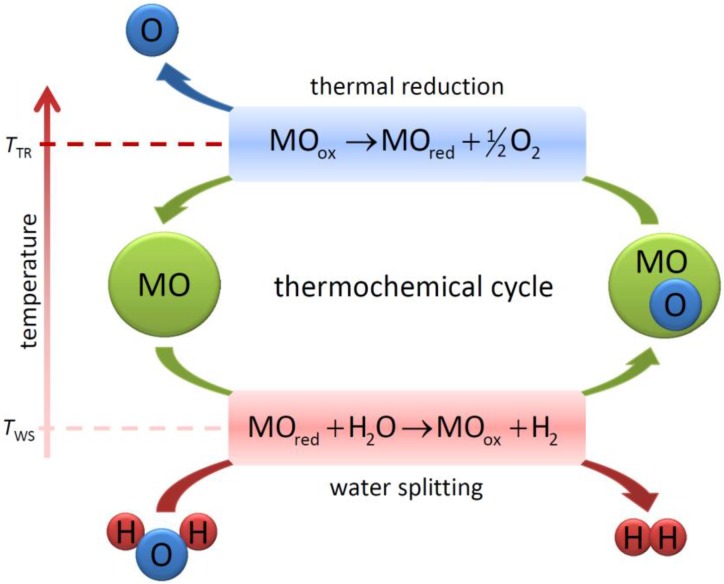
General schematic of the two-step thermochemical cycle for water splitting. MO denotes a metal-based redox material.

The concept of utilizing metal oxide-based redox materials in thermochemical two-step water-splitting cycles was first published in the late 1970s [17,18]. The general process concept is depicted in Figure 2.

MO denotes a metal-based redox material, which is either reduced (MO_red_) or oxidized (MO_ox_). In some processes, MO_red_ denotes an elemental metal. The first step is the solar-driven, endothermic dissociation of metal oxide either to the elemental metal or the lower-valence metal oxide. The water splitting is the exothermic hydrolysis of the reduced material to form H_2_. The overall reaction of the cycle is as follows:

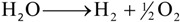
(1)
The process temperatures for each step strongly depend on the applied material and will be discussed in the following subsections. Typically, the thermal reduction takes place at much higher temperatures than the water splitting *T*_TR_ > *T*_W_ [13,16,19].

**Figure 3:**
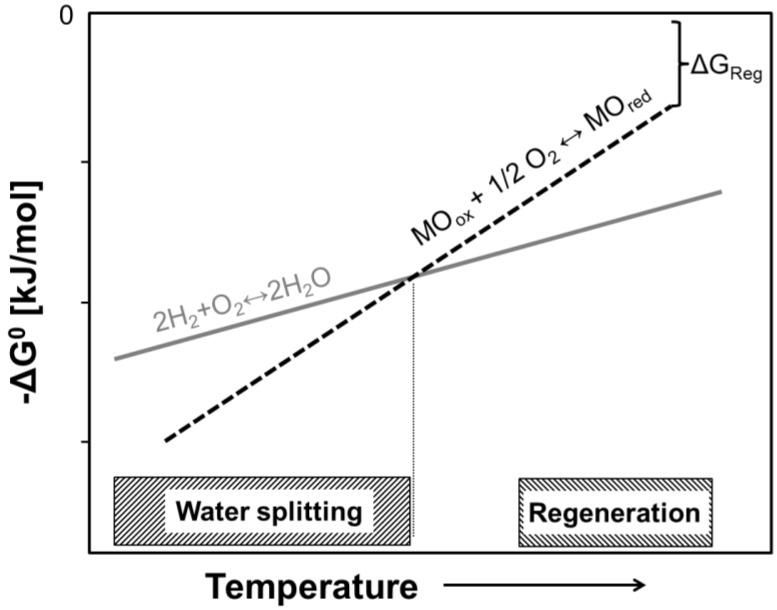
Schematic of free enthalpy of idealized MO_ox_ + 1/2 O_2_ ↔ MO_red_ redox reaction in comparison to H_2_/H_2_O equilibrium. Water splitting temperatures are below the intersection temperature. Regeneration is carried out at temperatures as high as technically feasible to reduce the energetic expense (∆*G*_Reg_).

Redox materials have to fulfill a wide range of properties. Thermodynamics is the *sine qua non*. It is a precondition for water splitting that the redox material in its lower oxidation state has a less noble character than hydrogen. For regeneration of the redox material, *i.e.*, for the reverse reaction from high to low oxidation state, the energetic expenses of the reverse reaction should be as low as possible. From the thermodynamic point of view, it is favorable to carry out the regeneration step at the highest temperatures (Figure 3).

**Figure 4:**
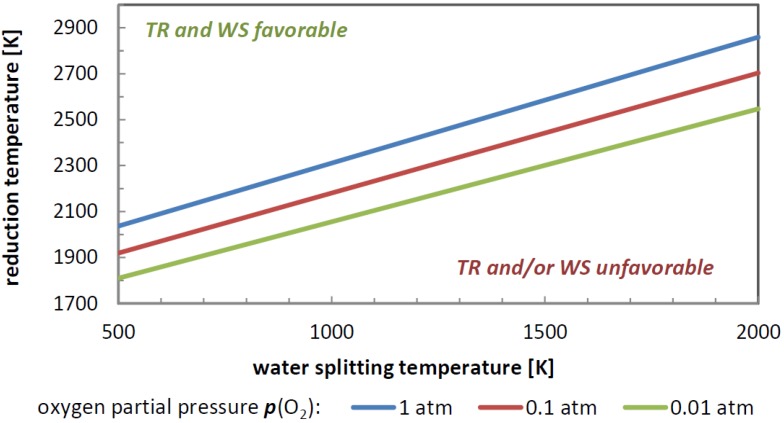
Temperature relation of the high and low temperature step depending on the pressure for which the reduction (TR) and the water splitting (WS) are thermodynamically favorable, neglecting solid-state entropy (no material-dependent properties are considered).

Neglecting the solid-state entropy of the materials, the minimum temperature difference of thermal reduction and water splitting ∆*T* is thermodynamically estimable [20]. At splitting temperatures of approximate *T*_WS_ = 973 K, the minimum reduction temperature is approximately 2173 K. In this regard, the driving force for the splitting cycle only consists of ∆*T* and of the entropy *S*_TR_(O_2_) of the released O_2_ at *T*_TR_. Fundamental thermodynamics also reveal the beneficial effects of lowering the partial pressure of oxygen *p*(O_2_) during reduction. The dependence of the process temperatures *T*_WS_ and *T*_TR_ at different *p*(O_2_) during reduction are depicted in Figure 4.

Considering solid-state entropy changes during the redox reaction (∆*S*_redox_ = *S*_red_ − *S*_ox_), the window of thermodynamically favorable *T*_WS_ and *T*_TR_ is broadened, if ∆S is large and positive. According to Figure 3, high entropy gain by transformation into the low oxidation state would be beneficial, because d*G*/d*T* corresponds to the reaction entropy. However, most redox material system exhibit negative entropy changes, making ∆*S*_redox_ an additional penalty for the thermodynamics of two-step water splitting cycles [20]. In addition to these thermodynamic considerations, the temperature ranges of a real two-step gas splitting reactor are restricted due to irreversible material degradation issues (*T*_TR_ > 1973 K) and due to very slow kinetics at low splitting temperatures (*T*_WS_ < 973 K).

High surfaces of the redox material, atomic mobility, the microstructural stability and the kind of solid-state transformation are key factors, as well. In case of reconstructive transformations between phases of different metal oxidation states, considerable nucleation barriers may occur resulting in a retardation of the redox reaction. Moreover, significant coarsening of a small-grained starting material will go along with the phase transformation if the nucleation density of the new phase is low. Certain multivalent metal oxide systems are beneficial in terms of nucleation since the change of oxidation states may be accomplished within one parent structure. A typical example is CeO_2_, which crystallizes in a fluorite-type structure and can be reduced up to CeO_1.65_ without structural breakdown of the fluorite base structure [21]. Another benefit correlated to nonstoichiometric defect structures is the fact that oxygen diffusion typically is high which facilitates the progress of the redox reaction from the surface to the bulk. A drawback from high atomic mobility, however, is rapid sintering and coarsening of the redox material. High surface areas are mandatory for the solid/gas redox reactions, but sintering effects facilitated by high atomic mobility counteract the small particle size of starting redox materials [22]. Thus, microstructural design strategies must be used to retain high surface areas of porous bodies or powder batches of redox materials. By segregation of solutes in grain boundary zones or by grain boundary precipitates, the mobility of grain boundaries can be affected and hence crystal and particle growth can be reduced [16,23]. Alternatively, the redox material can be applied as a coating on a stable substrate [24,25]. By using a counteracting substrate sintering shrinkage is reduced in two dimensions.

Using CO_2_ as the reactant gas instead of water steam, the metal oxide two-step redox cycle may also be employed for CO-production [22,26,27,28]. The production of fuel through the intermediate production of synthesis gas is discussed in the literature, as well [29,30,31,32,33].

The investigated reaction cycles might be technically classified as *volatile* cycles containing at least one gaseous species and *nonvolatile* cycles where all metal-based species remain in condensed state during the entire process.

### 2.1. Volatile Cycles

Volatile redox pairs employed in two-step water splitting cycles commonly exhibit a phase transition in the reduction step due lower boiling temperatures of the reduced species than the reduction temperature. The phase transition is thermodynamically beneficial for the process, because a high entropy gain is obtained. On the other hand, significant challenges occur due to recombination of the product gas stream, which implies either fast quenching of the gaseous species or a gas phase separation at high temperatures.

The main approaches that are recently discussed in literature are: the Zn/ZnO [19,31], the Cd/CdO [34,35] and the SnO/SnO_2_ [36,37] cycle.

#### 2.1.1. Materials

##### **Zinc** **(Zn)**

One of the most favorable volatile candidate redox pair for thermochemical water splitting is ZnO/Zn [19]:

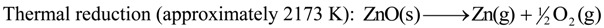
(2)

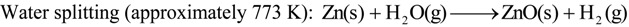
(3)
The reduction is highly endothermic and requires an energy input of 450 kJ/mol [38]. Reasonable dissociation rates of ZnO are achieved for temperatures above 2023 K. By lowering the reaction pressure and/or a carrier gas shift, the reaction is thermodynamically favored at lower temperatures. The exothermic splitting reaction releases 130 kJ/mol of energy and might be operated autothermally [38]. The exergy efficiency reaches 29% without any heat recovery. Assuming complete heat recovery during quenching and hydrolysis, an exergy efficiency of up to 82% is obtained [19].

Zn melts at 692 K and has a boiling point of 1180 K. Therefore after reduction, the product gas stream (Zn(g) + O_2_ + carrier gas) has to be quenched in order to separate O_2_ from Zn to avoid recombination. The quenching results in great technical challenges especially for the reactor design. The quenching consists of (1) diluting the Zn(g) and O_2_ in an inert flow gas (e.g., Ar) and (2) rapidly cooling the gaseous products below the Zn saturation and solidification points [31]. The separation efficiency depends on the dilution ratio and the temperature of the surface on which the products are quenched. For the technical realization, a quenching apparatus was suggested by Gstoehl *et al.* consisting of three distinct zones [39]. The first zone directly attached to the reactor exit exhibits temperatures above the ZnO decomposition temperature, thereby assuring that the product stream of Zn(g) and O_2_ enter the second zone without any recombination. In the second zone, an annular Ar flow suppresses the diffusion of Zn(g) towards the wall and the temperature is between decomposition temperature of ZnO and boiling temperature of Zn. For this temperature range, it was found that both products can coexist in a metastable state in the absence of nucleation site [19]. The actual quenching is proceeded in the third phase at temperatures below the Zn boiling point by an injection of Ar at cooling rates from 20,000 to 120,000 K/s suppressing ZnO formation in the gas phase and at the walls. For Ar/Zn(g) dilutions of 170 to 1500, conversions of 40%–94% were demonstrated [31]. Other concepts favoring a cooling lance to avoid recombination resulted in mean Zn yields of 8% for residence times of less than 1.8 s [38]. In lab-scale solar reactor testing at the solar furnaces and high-flux solar simulator of PSI/ETH (Switzerland) and CNRS-Odeillo (France), ZnO dissociation experiments of powders or pellets were performed. A 10 kW prototype reactor has been built (see Subsection: Technical implementation).

The splitting reaction towards H_2_, CO and syngas production was investigated by means of thermogravimetric analysis at temperatures of 623 to 1173 K. The reaction kinetics consist of an interface-controlled regime followed by a diffusion-controlled regime [40]. For the first five min of all experiments high reaction rates were observed followed by a constant rate. Zn nanoparticles were tested for water splitting in tubular aerosol flow reactor employing three steps: Zn evaporation, steam quenching and Zn/H_2_O reaction. The residence times were in the range of 1 to 2 s. At temperatures of 1023 to 1073 K, conversions from Zn to ZnO in the range 87%–96% were achieved [41].

##### **Tin** 

A novel two-step WS cycle based on tin oxide were proposed by Abanades *et al.* [36]:

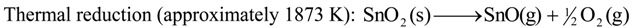
(4)

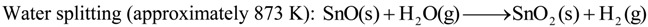
(5)
Due to the boiling temperature of SnO (*T_b_* = 1800 K), the reduction product is gaseous. Because of the recombination reaction, a quench is required to transform the gaseous SnO into nanoparticles as it is the case for the Zn/ZnO cycle. These particles are stable under ambient conditions and can be stored and transported easily. Thus, H_2_ generation on the delivery site on demand is possible.

Ideally, the intrinsic energy efficiency of the SnO_2_/SnO cycle is approximate 42% (reduction at 1873 K), which is similar to ZnO/Zn cycles at 2273 K. Compared to the ZnO/Zn cycle, the lower reduction temperature is advantageous, since it improves process sufficiency. When the gas temperature of the reduction product, SnO(g) + O_2_ is decreased, SnO particles rapidly condense because the gap between reduction temperature (1873 K) and SnO condensation temperature is small (SnO: *T*_m_ = 1315 K, *T*_b_ = 1800 K). The Zn/ZnO cycle is more dependent on a sufficient quenching, since the melting temperature is much lower (Zn: *T*_m_ = 693 K, *T*_b_ = 1180 K).

In lab-scale reduction experiments utilizing a 1 kW_th_ solar reactor illuminated by a 2 m-diameter parabolic dish concentrator, SnO_2_ to SnO conversions of 54% at atmospheric pressure were demonstrated [37]. The produced particles were recovered in a downstream filter after their entrainment by the inert carrier gas. The produced powder was typically composed of micron-size aggregates of nano-particles with diameters of 10–50 nm. Large specific surface areas of up to 60 m^2^/g were observed.

Thermogravimetric analyses of the SnO powders proved their capability for efficient hydrolysis as well as CO_2_ splitting. For water splitting, conversion rates approaching 90% were observed, while CO_2_ splitting requires a higher temperature for reaching the same conversion rate. From kinetic studies, two subsequent reaction regimes were identified. The first follows a first order reaction model. The second is limited by a diffusion mechanism at longer reaction times. This is similar to results of the zinc-based cycle [37].

#### 2.1.2. Technical Implementation

At the ETH Zurich a series of rotary reactors for the thermal dissociation of ZnO were developed, built and tested. [31,42,43,44,45] In these reactors ZnO particles are fed into a rotating cylindrical cavity. Through centripetal acceleration the ZnO particles are forced against the cavity wall. The particles are fed into the reactor with a special feeder system that can move inside the cavity thus enabling to spread the particles evenly along the cavity wall. The products (Zn vapor and O_2_) are carried out of the reactor through an Argon gas flow into the quenching zone, described in Chapter 2.1.

Based on economic analyses, the long-term potential of the Zn/ZnO-cycle realized in a solar tower system was determined. The H_2_ cost ranged between 0.10 and 0.15 $/kWh (based on its LHV and a heliostat field cost at 100 to 150 $/m^2^) [46].

A first process design study of the SnO/SnO_2_ thermochemical cycle was conducted at the CNRS. It features a solar concentrating system, a high-temperature solar reactor that will process the reduction, a water-splitting reactor, a quenching unit and a solid-gas separation. The reduction reactor operates either continuously or in semi-batch mode. To avoid recombination of SnO and O_2_, a quenching unit is connected to this reactor, recovering the sensible heat of the products that is transferred to the hydrolysis reactor. Combined with exothermal effects, the quenching process provides the energy needed for the hydrolysis [36].

### 2.2. Nonvolatile Cycles

Cycles employing nonvolatile redox pairs, which remain condensed during the whole process, bypass the recombination issue found for volatile cycles. Furthermore, these materials offer more possibilities concerning the reactor concept and process design for the reduction step due to the use of structured materials such as monolithic structures or coated foams, as well as the use of particle receiver. The main approaches will be introduced in the following.

#### 2.2.1. Materials

##### **Ferrites** 

The magnetite/wustite redox cycle was first proposed by Nakamura and one of the first materials used for solar-driven two-step water splitting [17]. In this cycle, water reacts with wustite (FeO) to form magnetite (Fe_3_O_4_) and hydrogen. In the next step magnetite is thermally reduced to form again wustite and oxygen according to the following reactions.


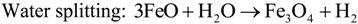
(6)


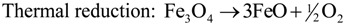
(7)

Thermodynamically, the first reaction, which is slightly exothermic, proceeds at temperatures below 1273 K at 1 bar. The thermal reduction, which is highly endothermic, proceeds at temperatures above 2573 K in air [16,47]. However, the temperature of thermal reduction is higher than the melting points of Fe_3_O_4_ (1808 K) and FeO (1643 K), resulting in a liquid Fe_3_O_4_ and FeO phase and causing a rapid decrease of the iron oxide surface area and thus a deactivation of the material [48,49]. The iron-oxygen phase diagram shows that a thermal reduction at 1623 K is possible if the oxygen partial pressure in the atmosphere is reduced to 10^−7^ bar [50]. But the production of such an atmosphere is rather energy-intensive and causes high process costs. Mixed solid solutions of Fe_3_O_4_/FeO and M_3_O_4_/MO (M = transient metal or alkaline earth metal) can be reduced at lower temperatures than the pure iron oxide system combining the high H_2_ yields of the Fe_3_O_4_/FeO system with the low reduction temperatures of a M_3_O_4_/MO system. In these systems, iron is partially substituted in Fe_3_O_4_ by a transition metal, e.g., manganese (Mn), cobalt (Co), nickel (Ni) or zinc (Zn), forming so-called mixed iron oxides or ferrites of the form (Fe_1-x_M_x_)_3_O_4_ while the reduced phase (Fe_1−*x*_M*_x_*)_1−*y*_O is still capable of performing the hydrolysis reaction. Numerous ferrites have been investigated in the past years with respect to their water-splitting capability.

In the 1990s Ehrensberger *et al.* studied the water-splitting reaction with mixed iron manganese oxides in a temperature range of 673–1173 K [51,52] and, about 10 years later, Mn_0.36_Fe_2.64_O_4_ was tested by Miller *et al.* [53]. Tamaura *et al.* studied the formation of oxygen-deficient ferrites between 1073 and 1373 K in a solar receiver-reactor configuration [54]. In these systems the phase transition between ferrite and wustite did not occur in the solid phase, but the ferrite retained its spinel-type crystal structure. Furthermore, studies were carried out on the formation of cation-excess magnetite and cation-excess Ni,Mn-ferrite [54,55]. During the reduction step an oxygen-free atmosphere is needed, and only relatively small amounts of hydrogen were generated in the water-splitting step due to the limited nonstoichiometry. Because of these drawbacks, no further investigations were reported.

Zn-ferrite ZnFe_2_O_4_ was intensively studied by Tamaura *et al.* between 2001 and 2005. ZnFe_2_O_4_ could be decomposed in a solar reactor under Ar atmosphere. Decomposition commenced at 1498 K and increased with increasing temperature, forming gaseous Zn, solid Zn*_x_*Fe_3−*x*_O_4_ and O_2_ [56]. In an air atmosphere, ZnFe_2_O_4_ could be decomposed at 1798 K, forming Fe_3_O_4_ and ZnO. The ZnO was separated from the magnetite and deposited on the reactor wall, suggesting that Zn vaporizes from the zinc-ferrite and recombines immediately with oxygen in air to form ZnO [57]. Mixtures of Fe_3_O_4_ + Zn and Fe_3_O_4_ + ZnO were investigated regarding their water-splitting ability. A mixture of Fe_3_O_4_ and Zn was able to produce hydrogen at a temperature of 873 K, forming Zn-ferrites (Zn*_x_*Fe_3−*x*_O_4_ (0.2 ≤ *x* ≤ 1)) and ZnO [58]. Fe_3_O_4_ mixed with ZnO was able to split water at 973–1073 K, forming a nonstoichiometric spinel product with lower zinc content than the stoichiometric ZnFe_2_O_4_ [59]. Nevertheless, the process was disregarded, because the separated ZnO and Fe_3_O_4_ must be collected and mixed after each thermal reduction step, which would result in a complicated reactor design and process operation.

In 2002, Kodama reported the use of Ni-ferrite in a methane reforming process combined with metal oxide reduction. Sintering of the Ni-ferrite occurred at high process temperatures, preventing a cycling of the redox-material. Ni-ferrite supported on zirconium dioxide (ZrO_2_) particles could be alternately reduced by methane and oxidized by steam, producing syngas and hydrogen respectively [60]. Based on these experiences, Kodama was the first to support Fe_3_O_4_ on zirconia particles for thermochemical water splitting to prevent sintering and deactivation of the redox material at high temperatures. The coated ZrO_2_ particles were tested and the cyclic reaction could be repeated at temperatures of 1673 K for thermal reduction and 1273 K for water splitting. ZrO_2_ alleviated the sintering of the solid reactant ferrite, and the phase transformation between Fe_3_O_4_ and FeO occurred on the crystals of ZrO_2_ [61]. Pure Fe_3_O_4_ as well as different ferrites of the form M*_x_*Fe_3−*x*_O_4_ were coated on ZrO_2_ particles (particle size of about 1 µm) and tested, (M = Mn, Co, Mg Ni) [49,62,63]. For comparison, unsupported pure Fe_3_O_4_, Co- and Mn-ferrites were tested, which converted to a nonporous dense mass during thermal reduction at 1673 K and could not be reused for subsequent water splitting [49,64]. Supported ferrites resembled an aggregation of fine sintered particles and were reused for water splitting after being pulverized again. NiFe_2_O_4_ was found to be the most suitable ferrite for water splitting, yielding high hydrogen production over repeated cycles. Nevertheless, the surface area was significantly reduced to about 3% of the original surface area during the first cycle but remained at the same order of magnitude during the subsequent cycles [49].

Kodama *et al.* also used Yttrium-Stabilized Zirconia (YSZ) as a support and found a new redox reaction occurring. When using zirconia doped with 3 mol-% of yttrium oxide (Y_2_O_3_) as a support for Fe_3_O_4_, a Fe^2+^-containing YSZ phase formed during the thermal reduction under an inert atmosphere at 1673 K, *i.e.*, the Fe^2+^ ions entered the cubic YSZ lattice. In the subsequent water splitting, Fe_3_O_4_ was formed again by reaction of Fe^2+^-YSZ with steam [65]. When YSZ doped with more than 8 mol % Y_2_O_3_ was used as a support, Fe ions remain in the YSZ structure. After the formation of the Fe-YSZ in the first thermal reduction step, the Fe^2+^ ions were oxidized to Fe^3+^ ions, remaining in the YSZ lattice during the water-splitting step. Increased the Y_2_O_3_ content in the YSZ stabilized the Fe^3+^-ions in the cubic crystal structure, thus Fe^2+^ ions could turn into Fe^3+^ at the lattice sites of YSZ [66]. A comparison of Fe_3_O_4_/YSZ and Fe_3_O_4_/ZrO_2_ showed that the YSZ-supported Fe_3_O_4_ with a high Y_2_O_3_content (Y_2_O_3_ > 8 mol % in YSZ) resulted in more stable hydrogen production than the ZrO_2_-supported Fe_3_O_4_. This was expected because FeO is not formed as the reduced phase in the system. Thus, melting of FeO and the scaling off from the YSZ support is prevented, due to remaining Fe^2+^ ions in the YSZ lattice throughout the repeated cycling [67]. Another comparison of Fe_3_O_4_ and NiFe_2_O_4_ supported on ZrO_2_ and on YSZ showed that NiFe_2_O_4_ supported on ZrO_2_ exhibit the highest hydrogen production rates during six cycles [68,69]. Nevertheless, the feasible combination of ferrite and support has not yet been found, and long-term tests concerning the stability have not yet been undertaken.

Coker *et al.* also studied in depth the reaction of iron oxide using YSZ as the support under thermochemical cycling using *in situ* technique (high temperature XRD and TGA) [70]. Furthermore, the oxygen permeation through the iron oxide/YSZ phase was investigated in order to understand the rate limiting processes of the reaction [71].

Ishihara *et al.* reported the formation of YSZ/Ni-ferrite and YSZ/Fe solid solutions. They performed 10 cycles in a laboratory reactor using YSZ/Ni-ferrite at a thermal reduction temperature of 1773 K in argon (Ar) and a water-splitting temperature of 1473 K [72,73,74]. They found that O_2_ and H_2_ outputs with the YSZ/Ni-ferrite solid solution were higher than those with Ni-ferrite and suggested that the oxidation and reduction of the iron ions proceed in the YSZ phase.

Miller *et al.* tested monoliths consisting of ferrite and YSZ phase assemblage. They used the robocasting technique, developed at Sandia National Laboratories, to fabricate monoliths consisting of a series of rods arranged in a face-centered, cubic-like geometry. Microscopic images showed that after testing, the ferrite grains within the structure were isolated from one another and were enclosed by YSZ. The exact reaction mechanism occurring during reduction and water splitting was not reported. A first material screening of Mn, NiMn, Ni and Co-ferrite powders showed Ni- and Co-ferrite to be very promising materials. They selected Co_0.67_Fe_2.33_O_4_ for the preparation of monoliths consisting of Co_0.67_Fe_2.33_O_4_ and YSZ (3 mol % Y_2_O_3_) in a 1:3 ratio [53]. Thirty-six cycles could be performed with a Co_0.67_Fe_2.33_O_4_/YSZ monolith at a thermal reduction temperature of 1673 K and a water-splitting temperature of 1373–1673 K. No significant degradation of the amount of hydrogen produced in a cycle was observed. For a comparison, a monolith consisting of pure Co_0.67_Fe_2.33_O_4_ was tested. Hydrogen could only be produced in the first cycle, confirming the necessity of YSZ to prevent deactivation of the ferrite [75].

Miller *et al.* substituted the YSZ by other supports such as aluminum oxide (Al_2_O_3_), titanium oxide (TiO_2_), hafnium oxide (HfO_2_) and yttrium-doped hafnium oxide (Y-HfO_2_). Monoliths consisting of Co_0.67_Fe_2.33_O_4_/Al_2_O_3_ and Co_0.67_Fe_2.33_O_4_/TiO_2_ produced only very small amounts of hydrogen during water splitting. Mixtures of Co_0.67_Fe_2.33_O_4_/HfO_2_ and Co_0.67_Fe_2.33_O_4_/Y-HfO_2_ did produce hydrogen during repeated cycling, but the hydrogen production was still smaller compared to the Co_0.67_Fe_2.33_O_4_/YSZ monolith. The support obviously plays an important role in both the reduction and the water-splitting reaction, but, as of now, the mechanisms of Co-ferrite and substrate interaction are not well understood [75].

Although first tests with the robocast monoliths were promising, a drawback of these structures is the very shallow light penetration into the reactive structure. Experimental results indicated that the material directly illuminated by solar radiation reduces more quickly than the material not directly illuminated, probably due to the slower rate of heat transfer to the interior of the structure via scattered thermal radiation, convection and conduction. Other reactive structures such as foams, vertical pins and textured plates are being developed, made of a mixture/slurry of the reactive material and YSZ [22].

Kodama tested coated foam devices consisting of magnesium oxide (MgO)-partially stabilized Zirconia (MPSZ) which were coated with Fe_3_O_4_/YSZ or NiFe_2_O_4_/ZrO_2_ particles [69,76]. Thirty-two cycles were performed with a Fe_3_O_4_/YSZ/MPSZ foam device at a thermal reduction temperature of 1673–1723 K and a water-splitting temperature of 1373 K. The foam device was broken after 32 cycles [77]. Twenty cycles were performed with a NiFe_2_O_4_/ZrO_2_/MPSZ foam, operating at temperatures between 1373 and 1723 K, and repeated hydrogen production was observed. The foam device remained, for the most part, intact but was slightly damaged at the edges after testing [68]. The MPSZ foam had a low thermal shock resistance, which led to damage of the foam during repeated thermal cycling. Also, large temperature gradients observed over the thickness of the direct irradiated foam due to the low heat transfer rates to the interior of the foam will lead to reduced activity of the colder sites of the foam and even completely inactive parts.

Scheffe *et al.* used cobalt ferrite CoFe_2_O_4_ deposited on Al_2_O_3_ supports via atomic layer deposition. Atomic layer deposition is used to form nano-scale films on particles of various substrates [78]. The technique was used to generate a material with high surface area. Multilayers of iron(III)-oxide and cobalt(II)-oxide were deposited alternately onto porous Al_2_O_3_ substrates, forming a film of 5 nm on the substrate. CoFe_2_O_4_ on Al_2_O_3_ was reduced at 1473 K, *i.e.*, at 200 K lower than CoFe_2_O_4_ coated on ZrO_2_, forming hercynite (FeAl_2_O_4_) and cobalt aluminate (CoAl_2_O_4_). It should be noted that this redox reaction intentionally incorporates the reaction between ferrite and support. Eight cycles were performed with CoFe_2_O_4_ on Al_2_O_3_ with nearly constant hydrogen production and no obvious degradation [79]. Recently, the amount of reactive material could be improved to about 20% CoFe_2_O_3_ on Al_2_O_3_ [80]. Furthermore, compared to bulk structures, extremely high reaction rates of Fe_2_O_3_ and Co*_x_*Fe_3−*x*_O_4_ ALD layers were demonstrated using ZrO_2_ as the substrate that preserved the high surface area [81].

Recently, a new concept based on metal oxides for solar thermal hydrogen production was investigated. The metal oxide is reduced thermally in air with concentrated solar energy. The hydrogen production step is conducted in an electrolytic cell using the reduced oxide either as an anode or solute in either an aqueous acid or base solution. The presence of the reduced metal oxide decreases the potential required for water electrolysis below the ideal 1.23 V. This concept was only investigated in a laboratory scale environment [82].

##### **Ceria** 

The ability of ceria to store and release oxygen in response to its environment is well known and this function has found utility in many applications including automotive catalysts, oxidation catalysts, reforming catalysts, and as an anode for solid oxide fuel cells. In 1985, Otsuka *et al.* proposed ceria as a water-splitting material [83]. Recently, ceria has attracted great attention, since it is one of the most promising materials being applied in two-step water-splitting cycles.

Ceria exists both in the +3 and +4 oxidation state. The complete redox cycle is shown here:

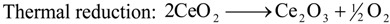
(8)

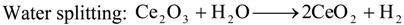
(9)


The solar-driven water splitting process utilizing ceria was firstly demonstrated at lab-scale by Abanades and Flamant [84]. The water splitting was studied in a fixed bed reactor. Fast kinetics could be demonstrated at temperatures of 673–873 K. The particle size does not influence the efficiency of the WS (diameter tested in the range 100–300 µm). XRD showed the complete conversion of the Ce_2_O_3_ into CeO_2_ due to the high reactivity of the reduced cerium oxide with water.

The thermal reduction was performed in a solar reactor at operating conditions of *T*_TR_ = 2273 K and *P*_TR_ = 100–200 mbar. These high temperatures required for the thermal reduction, result in practical problems of the reactor design due to the evaporation of ceria and high energy losses due to reradiation. Since the reduction process begins at temperatures of about 1673 K under oxygen deficient atmospheres, recent investigation are focused on the so-called partially-reduced ceria system, in which only a portion of the cerium atoms change their oxidation state:

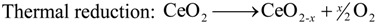
(10)

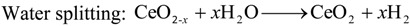
(11)


This partially-reduced ceria system maintains its fluorite-type structure up to *x* ≈ 0.35 [21], which is advantageous for its practical implementation in solar reactors as well as its results in rapid fuel production kinetics and high selectivity [22,85].

Due to the nonstoichiometric nature of this cycle, low specific H_2_ yields might be expected. However, specific yields of approximate 0.38–0.53 mmol H_2_ per gram of ceria were reported, which is competitive with that of other currently investigated thermochemical cycles [86] .

Ceria suffers from sintering at high temperatures. Combined with the fact that the oxygen storage and release is limited to the surface, the high temperature stability is identified as one of the major barriers to commercial success [22]. In order to overcome this drawback, the modification with another oxide is suggested. The modified materials are prepared via the combustion method [87] or the Pechini-derived polymeric route [85]. Doping with oxides that exhibit the same crystal structure is proposed to reduce the grain growth of ceria, as well as rendering the material easier to reduce. For example, zirconia is well known for decreasing the reduction temperature of ceria. This is due to the smaller size of Zr^4+^ than Ce^4+^ by which lattice deformations are introduced [28,88].

The formation of a solid solution between MO (M = Mn, Fe, Cu or Ni) and CeO_2_ was found to enhance the O_2_-releasing ability at lower temperatures compared to pure CeO_2_. Therefore, thermochemical two-step water splitting in a temperature range of 1273–1673 K was examined employing ceria doped with transition metals (Mn, Fe, Ni, Cu) [85,89]. Kaneko *et al.* obtained yields of 0.08 mmol H_2_ per g material for CeO_2_-MnO and CeO_2_-NiO. CeO_2_-Fe_2_O_3_ is significantly better than nondoped CeO_2_, whereas Cu does not improve the water splitting properties of ceria. Inversely, Abanades *et al.* reported for Al, Mn and Fe-doped ceria no reduction activity. Only for Co and Cu a reduction process were observed, which was ascribed to the reduction of CuO to Cu_2_O or Co_3_O_4_ to CoO, respectively. Reduced Cu- and Co-doped ceria powders, however, were not reactive with water at 1273 K. Only in the case of zirconia or yttria/zirconia-doping, did reduction result in Ce(III) species that were applicable for water splitting.

Le Gal *et al.* synthesized ceria-zirconia Ce_1−*x*_Zr*_x_*O_2_ solid solutions via different so-called soft chemistry routes in the range of *x* = 0 to *x* = 0.5 [88]. Two subsequent water-splitting cycles were carried out within a thermogravimetric balance. With increasing zirconia content, the reduction performance at 1673 K under an inert atmosphere was significantly improved. However, these results did not correlate to the water splitting performance at 1323 K. The highest amount of H_2_ in the second cycle was produced by the *x* = 0.25 powders in respect to the *x* = 0 and *x* = 0.5 samples. The authors concluded that moderate Zr contents favor H_2_ production during repeated cycles. Concerning the stability, the influence of the synthesis route was evidenced. For successive cycles, Zr-doped ceria synthesized via Pechini process yielded the largest amount of H_2_ and was found to obtain the most stable materials among the investigated synthesis routes [28].

##### **Perovskites** 

In the past years, a new thermochemical two-step water-splitting cycle emerged utilizing materials with a perovskite (CaTiO_3_) structure [90,91]. If suitable other cations are employed. Exsolution and re-incorporation of high amounts of oxygen may occur [92]. According to pulse reaction experiments, perovskites are expected to be applicable in redox processes for the dissociation of water [92].

Experimental studies on using perovskite-type materials for water-splitting cycles were conducted mainly by Evdou and Nalbandian [91,92]. They studied the redox potential of the perovskite materials of the general formula La_1-*x*_Sr*_x_*MO_3_ (M = Mn, Fe; *x* = 0; 0.3; 0.7; 1) by thermogravimetric oxidation/reduction experiments. In order to achieve a more effective and isothermal process, instead of using two-step thermochemical cycle, the partial oxidation of methane was employed, as shown here:

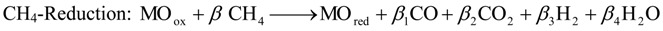
(12)


Thermogravimetry at 1173 K with alternating CH_4_ /He and O_2_/He input indicate that the materials are able to lose and uptake oxygen reversibly from their lattice up to 5.5 wt % for SrMnO_3_ and up to 1.7 wt % for LaFeO_3_ per minute. In terms of oxygen molecules released, this means 1.7 mmol O_2_ g^−1^ and 0.25 mmol O_2_ g^−1^, respectively.

Furthermore, the oxidation and reduction steps were combined in a novel membrane reactor constructed from dense perovskite membranes toward a continuous and isothermal operation (will be further explained in Section 2.2.2) [90,91].

#### 2.2.2. Technical Implementation

Until now only receiver-reactors that absorb the solar irradiation directly were developed for thermochemical cycles. Generally they can be divided into two types: One using particle streams and the other using solid reactive structures. The first concept is a volumetric gas particle solar receiver-reactor in which the reactive particles are directly irradiated, *i.e.*, a suspension of particles in a gas stream as, for example, in a falling particle film. Another possibility is to incorporate the redox material into solid structures that are then mounted in the reactor, e.g., coated foams or monolithic structures.

Two concepts exist to alternate the operating status of the reactants periodically, attaining either a continuous or a batchwise hydrogen production. For the former of the two reactor concepts, continuous H_2_ production is achieved by moving the reactive particles or structures from a thermal reduction reactor or zone to a water-splitting reactor/zone. The two reactors are operated at different temperatures; the thermal reduction reactor is heated by solar irradiation. Gas streams and solar irradiation can be provided continuously to the reaction chambers. The second concept foresees water splitting and reduction taking place in one single reaction chamber. By that means, batchwise production of hydrogen is achieved by switching the gas streams in the reaction chamber from reducing to oxidizing atmosphere. Simultaneously, solar flux densities need to be adjusted in order to realize the two different temperature levels and heat demands of reduction and water splitting. This is typically realized by diverting the solar flux periodically from one reaction chamber to the other.

A first reactor was tested at the solar furnace of the Paul Scherrer Institute (PSI) in Switzerland. It was a packed bed reactor consisting of a small quartz tube (2 cm diameter). The tube reactor was placed in the focus of the solar furnace, and a secondary concentrator was placed behind the reactor to provide a uniform irradiation of the tube. Ni_0.5_Mn_0.5_Fe_2_O_4_ powder mixed with Al_2_O_3_ grains was used as reactive particle bed. During thermal reduction, Ar was passed through the packed bed, and afterwards a mixture of Ar and steam was introduced. H_2_ and O_2_ evolution was observed [93], nevertheless, this concept was not followed up.

**Figure 5:**
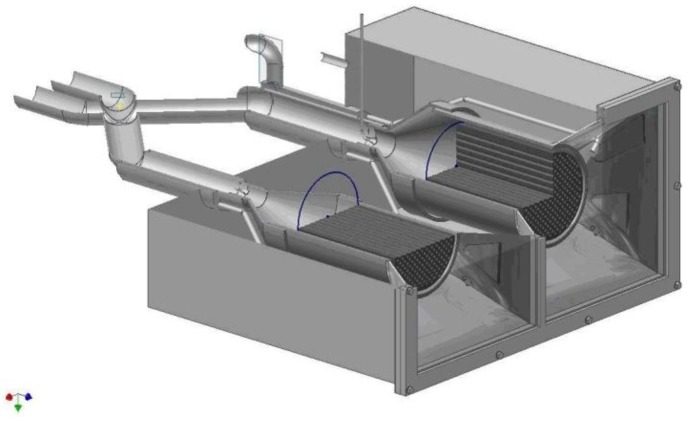
Solar reactor for quasi-continuous hydrogen production developed by the German Aerospace Center (DLR).

Several prototype reactors incorporating fixed coated ceramics have been developed in the past years at the German Aerospace Center (DLR) within the scope of the HYDROSOL project. The basic idea of the project was to combine a support structure capable of achieving high temperatures when heated by concentrated solar radiation, with a redox pair system suitable for the performance of water splitting and regeneration at these temperatures. With this idea DLR pursued the concept of a single solar receiver-reactor where the whole process (water splitting and regeneration of the metal oxide) takes place. Monolithic honeycomb structures made of siliconized silicon carbide (SiSiC) or recrystallized silicon carbide (ReSiC) were coated with a thin layer of mixed iron oxide and placed inside a solar receiver-reactor, where they served as the absorber for solar irradiation and provided the necessary surface area for the chemical reaction. A two-chamber reactor was developed that consisted of two adjacent but separated reaction chambers. In each reaction chamber a cylindrical honeycomb structure (Ø 144 mm) was placed as is seen in Figure 5. The concentrated solar irradiation enters the reactor through a quartz window and then impinges on the honeycombs, where it is absorbed. For quasi-continuous hydrogen production, one chamber is operated at about 1073 to 1273 K and flushed with steam, while the other is operated under a nitrogen atmosphere at 1473 K. The different heat demands of each step are realized in this process not by moving the reactors, but by periodically adjusting the solar flux on each reactor module when the status of the cycle is switched from regeneration to splitting and *vice versa*. This is done by a specific shutter system, the solar furnace allowing it to diminish the solar flux on each of the two chambers individually. For a plant on a solar tower, this is realized by periodical realignment of parts of the heliostat field. The reactor was tested at the solar furnace of DLR in Cologne [94,95]. A scaled-up reactor coupled to a solar tower system was developed with a thermal power input of 100 kWth. The tower reactor was installed on the Plataforma Solar de Almeriá (PSA) and consisted of two reactor modules. Nine square monoliths, each with an edge length of 146 mm, form one absorber module with an absorber surface of about 0.2 m^2^. The production of hydrogen was shown in several test campaigns [96].

Kodama *et al.* proposed an internally circulating fluidized bed reactor combined with a beam-down concept. In a beam-down concept or solar reflective tower the heliostat field illuminates a hyperboloidal reflector that is placed on a tower and directs the beams downward. The reactor would be built on the ground, and the solar irradiation enters the reactor through a quartz window in the ceiling. A circulating particle bed is created in the center and bottom region of the reactor. This design was chosen to prevent the particles from contacting the transparent quartz window. The reactor consists of a cylindrical reactor body. A draft tube is placed centrally in the fluidized bed region. Gases are introduced into the draft tube and the annulus region separately, thus creating a circulation of the particles. The particles are transported upward in the draft tube and move downward in the annulus region. The particle circulation within the reactor provides solar energy transfer from the top of the fluidized bed to the bottom region because directly irradiated particles of the top region move to the bottom region. A prototype reactor was built and tested using a concentrated Xenon (Xe)-lamp beam as a sun-simulator to heat the particles [63]. In the first tests, NiFe_2_O_4_/ZrO_2_ particles were thermally reduced in the reactor and the water splitting was conducted in an electrically heated fixed-bed reactor [97,98]. In the next development step, thermal reduction and subsequent water splitting were carried out in a single reactor heated by a concentrated Xe-lamp beam by switching the feed gas stream from N_2_ during reduction to a N_2_/H_2_O mixture for water splitting. Unsupported NiFe_2_O_4_ and supported NiFe_2_O_4_/ZrO_2_ were employed as redox material; the hydrogen production of the the NiFe_2_O_4_/ZrO_2_ being higher than that of the NiFe_2_O_4_ particles [64]. Since this reactor concept suffered from inhomogeneous irradiation distribution of the bed, only the center and top area reached the required 1673 K to be thermally reduced. The heat transfer inside the circulating particle bed was not sufficient to heat up the whole bed, which is necessary to gain fast and complete reduction of the whole bed.

The same research group proposed another reactor concept that incorporates coated foam devices. The foam device was placed on a fixed quartz plate inside the reactor and was directly irradiated by radiation from a Xe-arc lamp entering the reactor through a quartz window at the top of the reactor. The temperature of the foam device was controlled to about 1773 K at the center of the foam for performing the thermal reduction step. Water splitting took place in the same reactor by switching the gas supply from N_2_ to a N_2_/H_2_O mixture. The temperature was set to 1473 K during the water-splitting step. The reactor was tested with a MPSZ foam coated with NiFe_2_O_4_/ZrO_2_ (described in Chapter 2.2.1). Hydrogen was produced in six cycles, but large temperature gradients were observed in the foam [76]. A solar demonstration with a 5 kW_th_ dish concentrator with a coated MPSZ foam is planned to be tested at Inha University, Korea [68].

At the Tokyo Institute of Technology a rotary-type reactor was developed based on the concept of moving the reactants in order to continuously produce hydrogen. A rotary cylinder with reactive ceramics on its periphery is rotated in a cylindrical reactor chamber by an electric motor. The reactor contains two reaction cells: an oxygen releasing cell and a hydrogen generation reaction cell. By turning the rotor, each fraction of the reactive ceramic moves alternately through both reaction cells, allowing simultaneous and continuous evolution of O_2_ and H_2_. Ar is used in both cells as a carrier gas. To heat the reactive ceramics in the O_2_-releasing reaction cell, an infrared image lamp is used as a solar simulator with the irradiation passing through a quartz glass window in the reaction chamber [99,100].

Ni-ferrite, Ni,Mn-ferrite, CeO_2_ and CeO_2_-ZrO_2_ solid solutions coated on silica-mullite substrate were tested as reactive ceramics [101,102]. Hydrogen and oxygen production was achieved for six minutes, but reaction of Ni-ferrite with the substrate and sintering of the Ni-ferrite inhibited further continuous hydrogen production [103]. With YSZ Ni-ferrite, hydrogen was produced for about 15 min without degradation. The YSZ Ni-ferrite obviously reduced the sintering, and a layered design prevented the diffusion of SiO_2_ and Al_2_O_3_ into the ferrite [104].

Sandia National Laboratories has developed a solar-driven metal-oxide based heat engine, the Counter-Rotating-Ring Receiver/Reactor/Recuperator (CR5). The design allows for continuous H_2_ and/or CO, as well as O_2_ production. The reactor consists of several counter-rotating rings. On the circumference of these rings, fins made of reactive metal oxide are mounted. About one-quarter of the perimeter is illuminated by solar irradiation. As the rings rotate, they pass through the solar-irradiated, high-temperature zone where thermal reduction takes place. The water splitting happens on the opposite quarter of the perimeter. In the remaining two quarter sections countercurrent recuperation of heat occurs through thermal radiation. When the fins coming from the water-splitting section enter the recuperator section, they are heated up by the neighboring fins moving in the opposite direction, which simultaneously cool down [75,105]. The fins were robocast from ferrite and YSZ mixtures as reported in Chapter 2.2.1. Small-scale solar testing of Co-ferrite/YSZ robocast structures was performed with the solar furnace at Sandia National Laboratories. A robocast sample was tested at a thermal reduction temperature of 1853 K and a water-splitting temperature of 1323 K. Ferrite structures maintained their integrity for six cycles and hydrogen was produced [106]. Successful multiple full days on-sun testing of the CR5 is summarized in a recent Sandia report [107].

A reactor working with ceria was built and tested at the ETH Zürich. It consists of a cavity containing cylindrical rings made of porous ceria [108]. The concentrated irradiation enters the reactor through a window and is absorbed on the ceria inner walls. The gases are introduced radially flowing through the porous ceria into the cavity and exit the cavity axially at the bottom of the reactor. Temperatures up to 1913 K were used for the reduction phase while purging the reactor with argon. When cooling the reactor to about 1173 K, H_2_O or CO_2_ were introduced to investigate H_2_ and CO production. 23 cycles are reported for this reactor.

The University of Florida developed a reactor for an iron oxide based looping process. They used a magnetically stabilized fluidized particle bed reactor, in which a particle bed of iron powder mixed with silica powder with a volume ratio of 1:2 is filled and fluidized through an inert gas stream. Through a magnetic field the particle bed is stabilized through alignment of the iron particles along the field lines of the magnetic field. At elevated temperatures the iron particles sinter together and create a porous matrix of iron and silica. The water splitting step was conducted at 1073 K by introducing water vapor. For reduction of iron oxide back to iron, carbon monoxide was used that allowed reduction to proceed at 1073 K, as well—much lower than what would be required for thermal reduction [109].

A different reactor type was proposed by the Center for Research and Technology Hellas in Thessaloniki (Greece). They describe a perovskite redox membrane reactor working similarly to conventional electrochemical electrolyzer [90]. The proposed reactor consists of two compartments separated by a membrane made of an oxidic material. For water splitting, water vapor is introduced into one compartment. The material contains oxygen vacancies that react with water vapor to fill the vacancies with oxygen and simultaneously produce hydrogen. The lattice oxygen atoms are transported through the membrane to the other compartment that is kept at a low partial pressure of oxygen where they desorb to produce gaseous oxygen and leave oxygen vacancies. Another option stated is to introduce an oxidizable compound into the reduction compartment to enhance the creation of oxygen vacancies. Through the simultaneous oxidation and regeneration of the membrane, a continuous and isothermal process is obtained. A small scale reactor was built and tested with perovskite materials. Low hydrogen yields were achieved under unforced reduction conditions but could be significantly increased when reduction was enhanced with carbon monoxide.

## 3. Sulfur-Based Cycles

Many of the promising thermochemical cycles for water splitting use sulfur based systems. These cycles include the decomposition of the sulfuric acid which is carried out at a very high temperature level and in a corrosive atmosphere. Thus it constitutes the main issue in the coupling with any heat source. This reaction is usually carried out in several steps, between 573 and 1273 K.

### 3.1. Process Concepts

#### 3.1.1. Sulfur-Iodine Cycle

**Figure 6:**
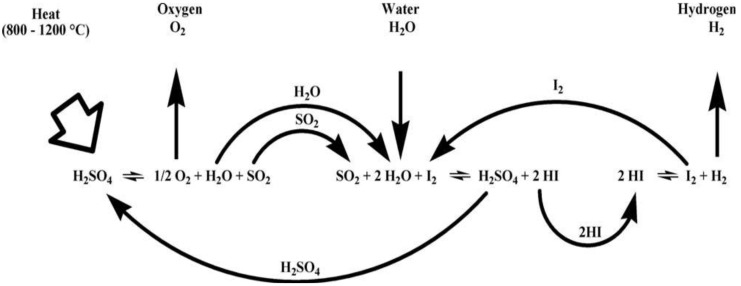
Sulfur-Iodine cycle.

The most investigated of Sulfur-based processes is the Sulfur-Iodine cycle, also known as ISPRA Mark 16 cycle or General Atomics Cycle (Figure 6).

It was originally proposed and developed in the USA by General Atomics in the mid-seventies [9].


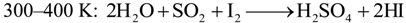
(13)


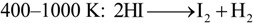
(14)


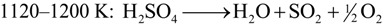
(15)

The Reaction (15) proceeds actually in two stages:

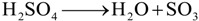
(15a)

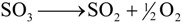
(15b)


The Bunsen Reaction (13) proceeds exothermically producing two immiscible aqueous concentrated acid phases. A lot of water and iodine are necessary in the Bunsen reaction to proceed and to obtain the two acids HI and H_2_SO_4_. The removal by distillation of the two acids requires a lot of energy. One of the major challenges of the sulfur-iodine cycle is to reduce these surpluses of water and iodine or to find separation processes consuming less energy than distillation.

The separation of HI from the Bunsen product mixture is complex and one of the most challenging tasks in the development of such process. The conventional distillation results in a distillate of azeotropic hydriodic acid and requires, in fact, large reboiler duty and, thus reducing the efficiency of the overall process [110]. To surmount this problem, General Atomics proposed an extractive distillation using phosphoric acid [111], while a reactive distillation at elevated pressure was proposed by Engels [112]. Up to now, the competition on which is the best solution for the HI separation appears to be still open.

HI is then decomposed according to Reaction (14) with a small endothermic heat and H_2_SO_4_ is decomposed according to Reaction (15), which is the major endothermic reaction of the cycle and takes place in the vapor phase in a catalytic reactor at about 1200 K.

The three sections of the process were built to demonstrate its feasibility in a glass, quartz and Teflon laboratory scale facility by JRC in Ispra, Italy [113]. Already in the eighties, the coupling to a solar thermal plant was designed. The process was improved at various locations like in the technical University of Aachen in Germany [114] or in Japan where JAEA has operated a closed-loop continuous hydrogen production at the rate of 32 L/h for 20 h in an experimental apparatus made of glass and fluorine resin [115].

The S-I cycle is, since the last decade, again in the focus of intensive research, not only because of the need to address the challenging reaction conditions, but mainly due to the perspectives created by the potentially high efficiency of this process. Several studies analyzed the thermal efficiency of this process. One of the most recent ones came up with a thermal efficiency between 33% and 36% based on the HHV of the product. This is considered as best estimate of the efficiency based on realistic assumptions for the unit operations of the process. An upper bound of the efficiency of 0.51 was found when assuming ideal behavior, such as entirely reversible reactions [116].

Concentrated sunlight can be considered as a suitable energy source to drive the sulfur-iodine cycle. The process was demonstrated on the solar power tower of the Georgia Institute of Technology [117,118], and in the solar furnace of DLR in Cologne, Germany [119], which also investigated the effective potential for massive hydrogen production of the sulfur-iodine cycle in the frame of the European projects Hythec [120]. Further lab to bench-scale demonstration studies were carried out by JAEA in Japan [115], by ENEA in Italy [121] and by CEA, Sandia NL and General Atomics in a common project in San Diego, USA [122].

#### 3.1.2. Hybrid Sulfur Cycle

**Figure 7:**
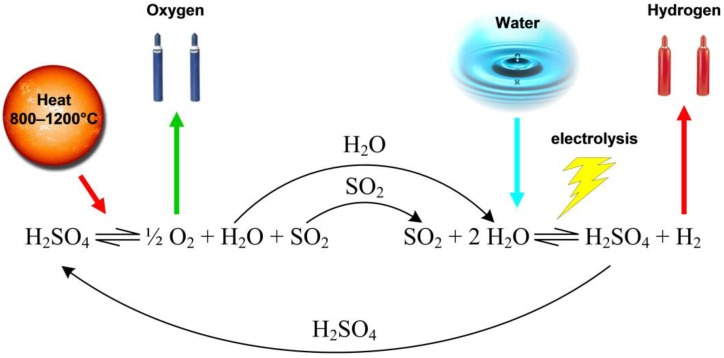
Hybrid Sulfur cycle.

The Hybrid Sulfur Cycle (Figure 7), also known as Westinghouse cycle or ISPRA Mark 11 cycle was originally proposed by Brecher *et al.* [123] and developed by the Westinghouse Electric Corporation in 1975.

It is a two-step thermochemical cycle for decomposing water into hydrogen and oxygen. It is a hybrid cycle because it combines the thermal decomposition of sulfuric acid with an electrochemical step which replaces the Bunsen reaction and the HI decomposition reaction of the sulfur-iodine cycle:

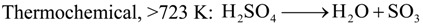
(16a)

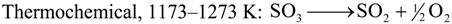
(16b)

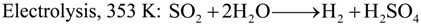
(17)

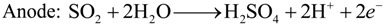
(18)

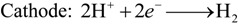
(19)


The voltage needed for the electrolysis is of 0.17 V and substantially lower than the 1.23 V needed for water electrolysis. Therefore the consumption of electrical power is significantly reduced. Sulfur dioxide and water are reacted electrolytically to produce hydrogen and sulfuric acid. The resulting sulfuric acid is decomposed in the same way as for the IS process, *i.e.*, it is vaporized to produce steam and sulfur trioxide, which is subsequently decomposed at high temperature into sulfur dioxide and oxygen. A catalyst is applied to accelerate the rate of sulfur trioxide reduction to sulfur dioxide and oxygen. This reaction is running better if the temperature is higher—namely, 1473 K—which can be reached with concentrated solar energy. There are several options to realize the sulfur dioxide/oxygen separation; most of them use a series of compression and cooling steps.

The advantage of this process is that it requires much less electric power than direct water electrolysis. Similar to the IS cycle, the hybrid sulfur cycle is predicted the potential for achieving high thermal efficiencies while using common and inexpensive chemicals [124]. Extensive work on electrolyzer and components development as well as on flow sheet modeling was carried out by Savannah River National Laboratory (SRNL) [124,125]. Process design and economic analyses to couple the hybrid sulfur cycle to a concentrating solar energy source have been presented by SRNL and CSIRO from Australia [126,127,128]. Predicted hydrogen production cost with such a process calculated within those studies go down to 3.19 and 5.57 US$/kg, respectively.

This cycle has been experimentally investigated at bench scale at the Research Center Jülich in cooperation with JRC in Ispra. Process Design, simulation and material investigation were performed in the frame of the EU projects HycycleS and HYTHEC to analyze reliability and potential hybridization of this cycle with solar energy [129], as well as to identify qualified materials for a technical realization of the solar thermal decomposition of sulfuric acid [130].

For SO_2_ electrolysis, carbon-supported platinum catalysts were employed to prepare the electrodes [131]. Ceramics such as silicon carbide, silicon nitrite, and cermet have an excellent resistance to H_2_SO_4_ corrosion at ambient temperature and at low acid concentration [132].

The process was developed actively by Westinghouse in the USA and in Italy by Euratom. A number of conceptual process designs lead to overall thermal efficiencies of up to 40% [132]. The product gas separation is suggested to be carried out by already established and technically mature methods [6].

#### 3.1.3. Outotec Process

The company Outotec has developed a new process to produce hydrogen and concentrated sulfuric acid (97–100 wt %) simultaneously from sulfur dioxide and water [133]. The process represents a combination of the generation of sulfuric acid from sulfur via the double contact process and the generation of hydrogen via the hybrid sulfur process or the sulfur-iodine process. In this so-called Outotec process, the sulfur dioxide stream is divided into two substreams. The first one (at least 40%) is mixed with water to enable the thermochemical or electrochemical production of hydrogen and sulfuric acid. The second one is oxidized over a V_2_O_5_ catalyst to sulfur trioxide (double contact process), which reacts with the water of the diluted sulfuric acid provided by the first substream to yield additional sulfuric acid and so achieves further concentration [134].

The hydrogen producing process steps are either the Bunsen reaction and HI decomposition according to the sulfur-iodine cycle or electrolysis of sulfurous acid according to the hybrid sulfur cycle. The coproduction of concentrated sulfuric acid and hydrogen introduces flexibility into the process and offers enormous economic advantages. If the sulfuric acid is used as a product recycling and decomposition of the acid is avoided. In that case, most of the energy needed to produce hydrogen is provided by the sulfuric acid production process.

#### 3.1.4. Others Processes

##### **HHLT Process** 

JAEA is developing a hydrogen production process, the thermochemical and electrolytic Hybrid Hydrogen process in Lower Temperature range (HHLT). It is a variant of the hybrid sulfur cycle, allowing operation at lower process temperatures. The HHLT process consists of the same reactions as the hybrid sulfur process.

In contrast to the hybrid sulfur process which needs a temperature of 1173–1273 K for the Reaction (16b), the HHLT process proceeds at a temperature of 773–823 K. For this, it employs two electrolyzers, one of which being a unique gaseous sulfur trioxide electrolyzer for the sulfuric acid decomposition reaction [135]:

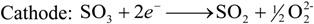
(20)

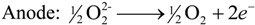
(21)


The process was demonstrated in a small lab-scale apparatus with a hydrogen production rate of 5 cm^3^/h for 60 h. The corrosion on high-temperature parts of the SO_3_ electrolyzer made of the material JIS SUS316, which is an austenitic stainless steel enhanced with an addition of 2.5% Molybdenum to provide superior corrosion resistance, covering with an anticorrosive layer of gold plating was less than expected. Some discoloration was observed on its inside but it stilled insignificantly and would not affect its operation. Therefore, in the next experiments, the SO_3_ electrolyzer will be made of JIS SUS316 without gold plating. Indeed, JIS SUS316 has improved pitting corrosion resistance and has excellent resistance to sulfates, and other salts as well, thereby reducing acids and a solution of chlorides, bromides and iodides [135].

The thermal efficiency reached in experiments was only 0.5%, but calculations suggested that the potential is much higher and can exceed 28% by adjusting the operating conditions and improving equipment performance, by optimizing the flow rate of sulfuric acid vaporization, by concentrating sulfuric acid with a multiple-effect evaporator and by reducing the cell voltages required for the electrolyzers. Detailed flow sheet evaluation is still necessary to determine accurate thermal efficiencies.

##### **Mark 13 V2 Process** 

The Mark 13 V2 process, which is a hybrid cycle, was developed by JRC in Ispra for coupling to a concentrating solar power plant.


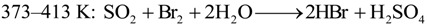
(22)


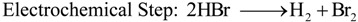
(23)


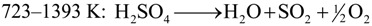
(24)

The use of Br_2_ leads to cycles in which the decomposition of the hydrobromic acid formed can be performed in an electrolytic cell.

A 1.4 × 10^6^ GJ/a solar power system was simulated. The calculated process efficiency of this cycle is 37% [136].

##### **Mark 13A Process** 

The Mark 13A sulfur dioxide cycle was developed by JRC in Ispra, as well. It is an enhancement of the Mark 13 cycle and based on two of its three reactions.


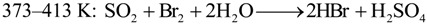
(25)


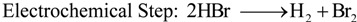
(26)

A complete bench-scale continuous process was successfully built and operated at Ispra. It was followed by the construction and operation of a pilot plant. This plant started regular operation in 1990 [137].

Both Mark 13 V2 and Mark 13 A processes are no longer studied. In comparison with the other known sulfur-based thermochemical processes for the production of hydrogen, the Sulfur-Iodine cycle and the Hybrid Sulfur cycle are the most favored ones. Among other reasons, this is mainly due to the following fact: the Mark 13 cycles are examples well suitable to demonstrate the problems of many thermochemical cycles. Although theoretically feasible, the removal of SO_2_ from the O_2_/SO_2_ stream in Reaction (24) is very difficult in terms of corrosion, efficiency, and cost. This separation is carried out in two steps. The first step consists of cooling the gas mixture to condense the bulk of SO_2_. In the second step, the remaining SO_2_ is removed from this gas mixture by contacting the gas with a dilute aqueous solution of bromine. SO_2_ is oxidized to sulfuric acid, which remains in the liquid phase, and the produced free oxygen contains only a few ppm of SO_2_. The formed HBr and H_2_SO_4_ are integrated in the cycle. This procedure leads to production costs for the process estimated at about 51 US$/GJ. This is about six times the calculated cost for hydrogen production via the Westinghouse cycle [138].

### 3.2. Materials

Similar to other thermochemical process also the sulfur based cycles require harsh operating environments for the efficient production of hydrogen and therefore a variety of material challenges exist. The wide range of process temperatures, pressures and the use of concentrated corrosive chemicals needs to be addressed by a careful choice of suitable materials or even by the development of new materials.

One of the key steps in the sulfur based thermochemical cycles is the dissociation of sulfuric acid into sulfur dioxide, oxygen, and water. The sulfur based cycles involve chemically aggressive chemicals and require material handling with high temperatures in excess of 1073 K and pressures along concentrated corrosive chemicals such as H_2_SO_4_ and its decomposition products. The combination of high temperature and highly corrosive chemicals imposes a specific demand on the stability and therefore choice of construction materials for some of the components. Within the European project HycycleS Alumina, Tantalum-coated steel and especially materials from the SiC-family have been investigated concerning their stability with respect to boiling concentrated sulfuric acid by carrying out long-term corrosion tests. Postcharacterization of the materials revealed that Siliconized SiC (SiSiC) was the most suitable one [139].

Within this project, DLR has worked on coupling the reactor for sulfuric acid decomposition with solar energy. Siliconized silicon carbide honeycombs coated with iron(III) oxide were prepared and tested in structured lab-scale reactors to evaluate their durability (*i.e.*, activity *vs.* time) during SO_3_ decomposition, with the result of satisfactory and stable performance for up to 100 h of operation [139].

Several series of corrosion tests were performed by General Atomics in order to select the best materials for the key parts of the S-I cycle. Specific emphasis was put on screening possible construction materials for the HI decomposition section—the one dedicated to perform Reaction (14). Selected corrosion-resistant materials were tested: refractory metals, coated metals, superalloys and ceramics. It was demonstrated that only Ta and Nb-based refractory metals and ceramic mullite can resist to the extreme HI*_x_* environment present in the HI section of the S-I cycle. Different zirconium-containing alloys, as well as a nickel-based superalloy C-276, which have excellent corrosion properties in a lot of applications, showed dissolution in the HI*_x_*-containing environment [140].

It was also shown that the utilization of surface alloying with tantalum via a specific method is very sufficient avoiding corrosion. The new tantalum surface alloying technology developed by the company Tantaline has the advantages of the superior corrosion resistance properties of Tantalum [141]. By diffusion of the tantalum atoms from a gaseous precursor into a substrate (typically stainless steel), a surface alloy is created. A dense layer of pure tantalum grows on the surface of the part over the diffusion layer, so that the treated substrate exhibits the same chemical properties and corrosion resistance as pure tantalum metal. General Atomics has used the tantalum surface alloys to build the key components facing environments containing iodine and hydriodic acid [141].

Further key materials for sulfur-based cycles are catalysts. The endothermic catalytic reduction of sulfur trioxide into sulfur dioxide and oxygen is the high-temperature step common to all sulfur-based thermochemical cycles. In order to obtain a satisfactory reaction rate in the sulfur trioxide splitting, catalyst systems featured by a high catalytic activity and a high stability under the harsh environmental conditions, need to be applied. It was shown that iron(III) oxide and related mixed oxides can be considered among the best catalyst options for the SO_3_ decomposition at temperatures equal or above 1123 K [142,143] and thus represent an inexpensive option for large-scale decomposer design. Such iron oxide-based alternative catalytic materials like spinels (AB_2_O_4_) or perovskites (ABO_3_) have been synthesized and evaluated [144]. Their catalytic activity was evaluated at 1123 K in a lab-scale apparatus. Fe_2_O_3_ catalysts have been coated on SiSiC honeycombs to be tested under realistic operating conditions with concentrated solar irradiation [145]. The catalyst stability was assessed after 100 h-long exposure to decomposition conditions at 1123 K. The results showed that Fe_2_O_3_-coated SiSiC honeycombs have the required specifications of an appropriate catalytic system, *i.e.*, that this catalyst ensured conversion efficiencies around 80% at the relevant boundary conditions, and exhibit only insignificant deactivation after exposure to the severe reaction conditions. Analyses have identified the presence of small amounts of sulfate on the catalyst surface, which have been formed during operation [145], suggesting that the catalysis mechanism proceeds via the formation of intermediate sulfate species.

## 4. Cu/CuCl Cycle

The main benefit of the copper-chlorine (Cu-Cl) cycle is its moderate temperature requirement compared to other processes. The highest temperature needed in one of the process steps is 800 K. Moderate process temperature offers the potential use of low-grade waste heat to improve energy efficiency, and potentially lower cost materials. The voltage required for the electrochemical step and thereby electricity input is relatively low.

The main challenges of the process are the intermediate product treatment and separation and to construction materials. Solids handling between processes and corrosive working fluids, however, pose unique challenges. The development of corrosion resistant materials for these working fluids is necessary.

Using intermediate copper chloride compounds, the cycle decomposes water into hydrogen and oxygen, in a closed internal loop that recycles all chemicals on a continuous basis. The chemical reactions making up the Cu-Cl cycle in its best known process variant are listed as follows.

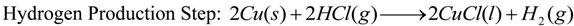
(27)

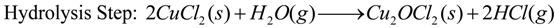
(28)

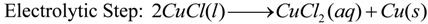
(29)

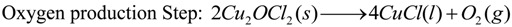
(30)

In the first step, hydrogen chloride gas and solid copper react together to form liquid copper chloride and hydrogen, the target product of the cycle. The reaction is exothermic and takes place at a temperature in the range of 703 to 748 K.

The hydrolysis step is endothermic and is operated at temperatures in the range of 648 to 673 K. The reaction takes place between solid copper chloride and water steam. The products of this step are hydrochloric gas und copper oxychloride.

The voltage needed for the third step, the electrolysis of copper chloride solution at ambient temperature, is substantially less than needed for water electrolysis: in between 0.4 and 0.6 V.

The last step represents the release of oxygen. Copper oxychloride decomposes under heat supply to oxygen gas and liquid copper chloride.

University of Ontario Institute of Technology (UOIT) in Canada is the institution most active on this cycle and on all related investigations on process and material development, as well as on process integration and economic evaluation. Other players in this field are Atomic Energy of Canada Limited (AECL) and Argonne National Laboratory (ANL). Proof-of-principle experiments for each of those steps have been conducted [146,147]. The experimental results have been reported for most of the processes of the Cu-Cl cycle, and large laboratory scale reactors for the processes have been tested successfully. A one-third overall thermal efficiency improvement over alkaline electrolysis is predicted by process simulation, if “waste heat” is utilized in the CuCl cycle. A heat to hydrogen (LHV) cycle efficiency of 43% was predicted, when assuming realistic heat exchanges efficiencies, which compares to 31% for alkaline electrolysis if assuming the same boundary conditions [148].

### 4.1. Process Concepts

There are three key variations of the CuCl cycle: 5-step, 4-step and 3-step cycles. In the 5-step cycle, copper is produced electrolytically, moved to an exothermic thermochemical hydrogen reactor and then reacted with HCl gas to produce hydrogen gas and molten CuCl. The 4-step cycle combines these processes together to eliminate the intermediate production and handling of copper solids, through a CuCl/HCl electrolyzer that produces hydrogen electrolytically and aqueous Cu(II) chloride. The latter product is dried to generate Cu(II) chloride particles, then fed to a hydrolysis reactor to produce copper oxychloride. The three-step cycle further combines these processes by supplying aqueous Cu(II) chloride directly into the hydrolysis chamber, such as spraying the solution with co-flowing steam to produce the same copper oxychloride product.

The advantages and disadvantages of those process variants have been discussed in detail [149]. In general, decreasing the number of steps requires either higher process temperatures, or application of electricity-intensive electrolysis step. Increasing the number of steps, on the other hand, means to have more equipment, more material to be recycled and more challenging separation issues.

**Figure 8:**
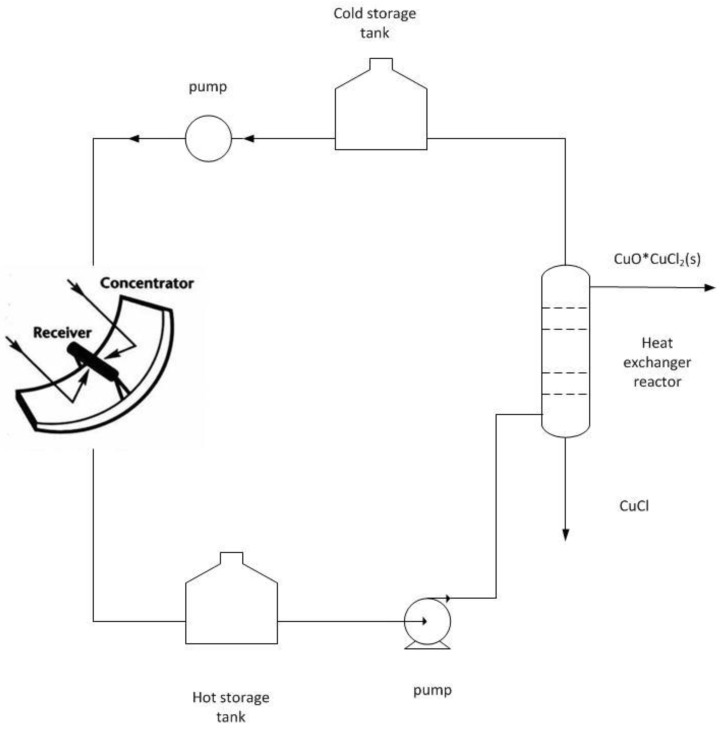
Schematic of a plant for Cu_2_OCl_2_ decomposition using molten salt storage medium.

Whereas the main focus of this cycle was the potential coupling to a nuclear reactor and thus most of the studies published elaborate on this, also solar concentrating systems are considered as a potential energy source to supply the necessary heat and electricity of this cycle. The last step of the CuCl cycle, the oxygen generation reactor, is predestined to be coupled to a solar heat source due its high temperature heat requirement. As a representative and promising scenario, the coupling of this reactor to a solar trough plant with molten salt as working medium has been proposed and analyzed [12]. The coupling scheme is depicted in Figure 8.

The concept foresees a linear parabolic collector system with suitable thermal storage capability to compensate the solar input variability. A mixture of molten salts is used as the heat transfer fluid and storage media. Such molten salts are e.g., currently used in some commercial solar power plants like the GemaSolar Plant or the Andasol Plants in Spain [150,151]. Similar to the process in those plants, here a binary mixture of 60% NaNO_3_ and 40% KNO_3_ was considered. Besides the collectors’ storage tanks, circulation pumps and a heat exchanger reactor designed and selected for operation with hot salt are needed. The molten salt heated in the collector to more than 823 K is collected and sent to a hot reservoir for heat storage. On demand, the hot salt is pumped to the chemical process and cooled by the endothermal reaction to lower temperature levels. After cooling, the molten salts are sent to the cold tank and pumped to the parabolic troughs to restart the heat-collection loop. The heat exchanger reactor is a countercurrent type with molten salts on the shell side. Heat is transferred to the inner tubes where the reactant flows and reacts to yield oxygen and CuCl(l). The study showed that for selected sites solar-based thermochemical production of hydrogen by the CuCl cycle is feasible on a large scale.

### 4.2. Materials

There are a number of aspects related to materials investigation and development worked on for the CuCl cycle. The first aspects deals with the hydrolysis of Cu(II) chloride. Transport phenomena associated with the gas-solid reaction of particles in the fluidized bed during hydrolysis of Cu(II) chloride were addressed by both numerical and experimental investigations. The conversion of solids depends on the reaction rate and residence time of a particle. When the diffusion of gaseous reactants into a particle is much faster than the chemical reaction, the solid reactant is consumed nearly uniformly throughout the particle. Therefore a uniform reaction model can display the reaction. When the diffusion of gaseous reactant is much slower and it restricts the reaction zone to a thin layer that advances from the outer surface into the particle, the shrinking-core model must be adopted. In case of the hydrolysis step of the Cu/CuCl cycle a shrinking-core model turned out most appropriate and has been applied to determine the reaction rate of the gas-solid reaction [152].

Construction materials are an issue as well since some of the process steps use components which are exposed to corrosive environments [153]. Material degradation studies were performed for selected materials including metals, ceramics, elastomers, polymers, carbon-based and composites under the expected operating conditions of the CuCl/HCl electrolyzer [153]. Each was exposed to operating conditions of 433 K, 2.5 MPa and concentrated solutions of HCl, CuCl, and CuCl_2_. These very aggressive conditions accelerate the corrosion reactions. The electrolyzer requires a wide range of construction materials. Glass-lined metal turned out not to be suitable, since it was found that glass dissolved up to 0.7 mm/year under aqueous conditions.

Researchers at UOIT analyzed and qualified ceramic carbon electrodes (CCE) for the anode of the CuCl/HCl electrolysis cell [154]. The CCE catalyst layer is a three-dimensional porous structure composed of carbon black and poly aminopropyl siloxane. Additional experiments investigated the CCE performance at higher concentrations of CuCl [155]. A clear performance advantage is retained at high CuCl concentrations which are highly representative for the targeted cell operating conditions.

Nickel alloy coatings were also developed for corrosion resistance against high temperature Cu(I) chloride and HCl in the hydrolysis reactor. In experimental studies, various surface coatings were evaluated. The corrosion performance of materials was tested by immersing coupons in CuCl/HCl containing environments. It was found by impedance spectroscopy that Inconel 625 has a higher imaginary impedance and resistance than Al6XN stainless steel, suggesting that the naturally formed oxide scale on Inconel is more protective than the passivation layer formed on Al6XN.

## 5. Conclusions

It was analyzed and depicted that for all relevant processes applicable for high temperature solar-chemical hydrogen production by multistep water spitting, materials play a key role, which is due to the strict and narrow requirements of the process conditions and the need for efficient and economic operation impose on those materials. A key task for many processes is the identification of suitable redox and catalyst materials exhibiting not only sufficient reactivity and activity, respectively, but also sufficient microstructural stability over the long term, and over many cycles of operation.

Suitable materials for substrates and containments for high temperature water-splitting processes require being stable against the relevant reaction systems and the often corrosive reaction environments have been investigated and many cases have been identified. This also had to take into consideration their solar absorbance and thermo-shock resistance. Several approaches have been suggested for the solar interfaces, which ensure, on the one hand, the necessary process temperatures and that the processes are supplied with the required amount of heat and, on the other hand, that the available concentrated solar energy is efficiently used. To match the materials properties required for the reaction with the properties of the solar interface needed for an effective collection of the available energy is and remains one of the key challenges.

Metal–metal oxides pairs and multivalent metal oxides are the basic materials for so-called redox cycles. The most promising material families are based on rare earth metals or ferrites. A number of process concepts have been introduced to integrate such cycles in a solar plant. Some are based on fixed or moving particle technologies such as fixed beds, fluidized beds, rotary kilns; others use monolithic bodies such as honeycomb structures or foams made of or coated by the active redox material. Besides the required high process temperatures and separation issues, the main challenge of those processes is the availability of sufficiently active and stable redox materials.

Sulfur-based cycles represent processes that comprise as one central step the industrially widely established decomposition of sulfuric acid, coupling in concentrated solar energy at temperatures significantly below 1200 K. As the most prominent examples of sulfur-based cycles, the sulfur-iodine cycle and the two-step hybrid sulfur cycle are particularly promising, since they exhibit low specific hydrogen costs at high efficiencies. All reaction steps have been successfully demonstrated and reached a level of maturity sufficient for the development of a pilot plant. Further improvements of catalyst and construction materials need to be addressed for an industrial realization. This is in particular true for a key step, which all those sulfur-based processes have in common: the high temperature decomposition of sulfuric acid.

The four-step version of the copper chlorine cycle represents a promising alternative due to the fact that all process steps take place at lower temperatures compared to other thermochemical cycles. In addition to that, the electrochemical step of this cycle only require low voltage challenges still have to be addressed concerning reaction selectivities and product separation.

Solving the main materials-related issues and providing the right functional materials at reasonable costs will decisively help solar thermal processes to achieve the role of significant contributors to carbon-free and sustainable hydrogen and syngas production on a large scale, using only solar energy, carbon dioxide and water as clean and abundant sources for those fuels.
